# Enhanced Microbial Survivability in Subzero Brines

**DOI:** 10.1089/ast.2017.1805

**Published:** 2018-09-12

**Authors:** Jacob Heinz, Janosch Schirmack, Alessandro Airo, Samuel P. Kounaves, Dirk Schulze-Makuch

**Affiliations:** ^1^ Center of Astronomy and Astrophysics, Technical University of Berlin, Berlin, Germany.; ^2^ Department of Chemistry, Tufts University, Medford, Massachusetts.; ^3^ Department of Earth Science and Engineering, Imperial College London, London, United Kingdom.; ^4^ School of the Environment, Washington State University, Pullman, Washington.

**Keywords:** Brines, Halophile, Mars, Perchlorate, Subzero, Survival

## Abstract

It is well known that dissolved salts can significantly lower the freezing point of water and thus extend habitability to subzero conditions. However, most investigations thus far have focused on sodium chloride as a solute. In this study, we report on the survivability of the bacterial strain *Planococcus halocryophilus* in sodium, magnesium, and calcium chloride or perchlorate solutions at temperatures ranging from +25°C to −30°C. In addition, we determined the survival rates of *P. halocryophilus* when subjected to multiple freeze/thaw cycles. We found that cells suspended in chloride-containing samples have markedly increased survival rates compared with those in perchlorate-containing samples. In both cases, the survival rates increase with lower temperatures; however, this effect is more pronounced in chloride-containing samples. Furthermore, we found that higher salt concentrations increase survival rates when cells are subjected to freeze/thaw cycles. Our findings have important implications not only for the habitability of cold environments on Earth but also for extraterrestrial environments such as that of Mars, where cold brines might exist in the subsurface and perhaps even appear temporarily at the surface such as at recurring slope lineae.

## 1. Introduction

Life as we know it requires liquid water as the principal solvent for its biochemistry, but most planetary surfaces in our Solar System never reach temperatures above the freezing point of pure water, rendering these localities as likely uninhabitable compared with the benign climate conditions on Earth. However, the presence of salts can lead to a substantial freezing point depression down to the eutectic temperature of a given salt/water mixture (*e.g.*, −50°C for a 31 wt% CaCl_2_ solution) and, thus, greatly expand the temperature range for potential habitats (Möhlmann and Thomsen, 2011). Hence, the question arises as to whether microorganisms can thrive or at least survive in such subzero brines.

On Earth, microbial organisms such as yeast can tolerate water activities (a_w_) down to 0.61 (Rummel *et al.*, 2014). However, the lowest salt-induced water activity that halophilic microorganisms can tolerate is that of a saturated NaCl solution (a_w_ = 0.75), while other salts (*e.g.*, those containing Ca^2+^ and Mg^2+^ ions) are more inhibitory to cell metabolism (Rummel *et al.*, 2014). Furthermore, it has been reported that certain cyanobacterial species embedded in hygroscopic sodium chloride (NaCl) deposits found in the hyperarid soils of the Atacama Desert are able to utilize water condensed from the atmosphere via deliquescence (Davila *et al.*, 2008; Davila and Schulze-Makuch, 2016).

In addition, many halophilic microorganisms can also be psychrophilic or psychrotolerant (Gounot, 1986; Hoover and Pikuta, 2010). To date, the lowest reported temperature for microbial growth is −18°C for yeast on frozen surfaces (Collins and Buick, 1989). Metabolic ammonia oxidation has been detected down to −32°C (Miteva *et al.*, 2007), and finally, there are indications for photosynthetic activity of lichens at −40°C (de Vera *et al.*, 2014).

It has been argued that low-temperature and high salt tolerances are closely linked, given that at subzero temperatures, water ice forms, which increases the solute concentration of the remaining liquid water (Bakermans, 2012). Moreover, chaotropic agents such as magnesium chloride (MgCl_2_), that is, substances that destroy the bulk water structure and therefore reduce hydrophobic interactions (Gerba, 1984) at moderate concentrations, can decrease the minimal temperature at which cell division can occur for certain microorganisms and increase their growth rates at low temperatures, presumably by increasing the macromolecular flexibility (Chin *et al.*, 2010). Furthermore, some microorganisms shift their salinity optimum for growth to higher salt concentrations if exposed to lower temperatures (Gilichinsky *et al.*, 2003).

Organisms have evolved several adaptations for thriving and/or surviving in cold saline environments. These include production of antifreeze or ice-binding proteins, cryoprotectants, or extracellular polymeric substances (Jia *et al.*, 1996; Gilbert *et al.*, 2005; Kuhlmann *et al.*, 2011), an increase of fatty acids that branch and maintain membrane fluidity (Denich *et al.*, 2003), a higher antioxidant defense against reactive oxygen species (Chattopadhyay *et al.*, 2011), the expression of isozymes adapted to low temperatures and high salinities (Maki *et al.*, 2006), or the exclusion of inhibitory ions by accumulating intercellular compatible solutes (Csonka, 1989).

Most of the studies dealing with brines at subzero temperatures have focused on NaCl as a solute, the most common salt found in saline environments on Earth. However, certain environments on Earth are dominated by high concentrations of other salts such as calcium chloride (CaCl_2_) in Don Juan Pond, Antarctica (Cameron *et al.*, 1972; Dickson *et al.*, 2013), or sodium and magnesium sulfates in Spotted Lake, Canada (Pontefract *et al.*, 2017). Furthermore, martian soils are known to contain various chloride (Cl^−^) and perchlorate (ClO_4_^−^) salts (Hecht *et al.*, 2009; Kounaves *et al.*, 2010), emphasizing the importance of research in the field of non-NaCl briny habitats at subzero temperatures.

In this study, we used the halo- and cryotolerant bacterial strain *Planococcus halocryophilus* Or1 (DSM 24743^T^) isolated from the active layer of permafrost soil in the Canadian High Arctic (Mykytczuk *et al.*, 2012). This organism grows at temperatures between −15°C and +37°C and under NaCl concentrations of up to 19 wt% at which metabolic activity has been detected at temperatures down to −25°C (Mykytczuk *et al.*, 2013).

This bacterial strain shows many cold and osmotic stress responses such as the expression of cold-adapted proteins, the expression of various osmolyte transporters, a high lipid turnover rate, a high resource efficiency at cold temperatures with an accumulation of carbohydrates as a energy resource (Mykytczuk *et al.*, 2013), and complex changes in protein abundances (Raymond-Bouchard *et al.*, 2017). Furthermore, under cold growth conditions, *P. halocryophilus* develops a nodular sheet-like crust around the cells (Ronholm *et al.*, 2015; Mykytczuk *et al.*, 2016).

The above-described ability of *P. halocryophilus* to cope with low temperatures and high salt concentrations makes it an ideal organism for studying whether, and how well, terrestrial life might be able to survive or even thrive in martian environments. In particular, we have investigated how well *P. halocryophilus* can survive repeated freezing/thawing cycles and in subzero chloride and perchlorate brines, since such conditions may be temporarily present on Mars (Martínez and Renno, 2013).

## 2. Materials and Methods

### 2.1. Strain and culture conditions

**FIG. 1.**
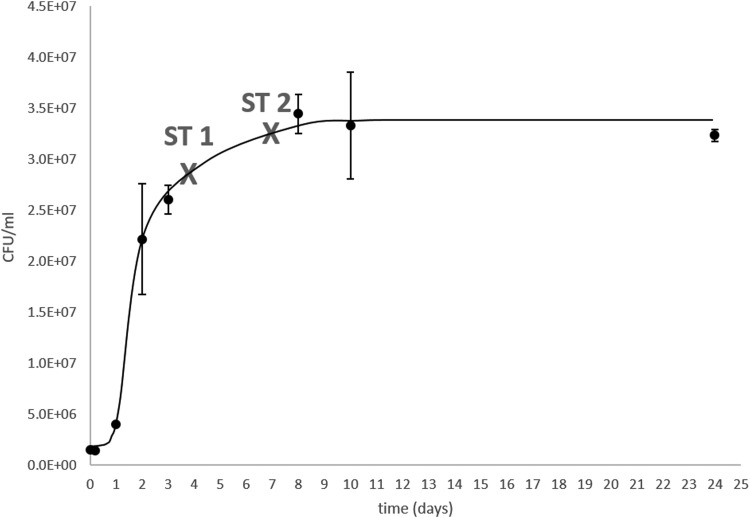
Bacterial growth curve of *Planococcus halocryophilus* in DMSZ growth media #92 + 10 wt% NaCl at 25°C. CFUs obtained as technical duplicates. Crosses mark the sampling times for inoculating of sample types ST 1 and ST 2. CFUs, colony forming units.

We used the bacterial strain *Planococcus halocryophilus* Or1 (DSM 24743^T^), which was obtained from the DSMZ (Leibniz Institute DSMZ—German Collection of Microorganisms and Cell Cultures). *P. halocryophilus* was grown in DMSZ growth medium #92 containing additional 10 wt% NaCl. Its growth curve at 25°C was determined via colony forming units (CFUs), and cell suspensions used for inoculating the experiments were either retrieved after 4 days (sample type ST 1) or 7 days (sample type ST 2) of growth (Fig. 1).

### 2.2. Experiments in eutectic salt solutions

In all experiments, 2 mL of the cell suspension (prepared as described in Section 2.1) was mixed with 8 mL of a salt solution resulting in 10 mL of sample solution with a eutectic salt concentration. The eutectic compositions of the investigated salts are listed in Table 1, together with the ionic strength, the water activity at 25°C calculated from the Pitzer equation (Pitzer, 1991) with Pitzer parameters taken from the work of Toner *et al.* (2015), and the eutectic temperature. All samples were prepared and analyzed as biological duplicates.

**Table 1.** Eutectic Concentrations and Temperatures, Ionic Strength, and Water Activities at 25°C for Salt Solutions Used in This Study

	*Eutectic concentration*		*Ionic strength*	*Water activity at 25°C*	*Eutectic temperature*	
	*wt%*	*mol/L*	*mol/L*		*°C*	*K*
NaCl	23.3^a^	5.20	5.20	0.80	−22	251^a^
MgCl_2_	21^a^	2.79	8.38	0.75	−33.5	239.5^a^
CaCl_2_	30.2^a^	3.90	11.70	0.65	−50	223^a^
NaClO_4_	52.6^b^	9.06	9.06	0.68	−34	239^b^
Mg(ClO_4_)_2_	44^a^	3.52	10.56	0.56	−57	216^c^
Ca(ClO_4_)_2_	50.1^d^	4.20	12.60	0.52	−77.5	195.5^d^

^a^ Möhlmann and Thomsen (2011).

^b^ Hennings *et al.* (2013).

^c^ Stillman and Grimm (2011).

^d^ Pestova *et al.* (2005).

Before mixing cell suspensions and salt solutions, the suspensions were cooled to 4°C and the salt solutions to the respective experimental temperature. In addition, for testing whether ClO_4_^−^ preconditioning of the cells has a positive effect on their survival in ClO_4_^−^ containing samples, cell suspensions with either up to 10 wt% NaClO_4_ or 5 wt% NaClO_4_ + 10 wt% NaCl were prepared and incubated for 7 days at 25°C.

### 2.3. Cell number quantification

The concentration of viable cells in the samples was determined after specific time intervals via CFU counts, and, where necessary, samples were diluted in phosphate-buffered saline (PBS) containing 21 wt% NaCl or MgCl_2_ to avoid osmotic bursting of cells. Highest values of CFU mL^−1^ were achieved when dilution was done with NaCl-enriched PBS for samples containing NaCl, MgCl_2_, NaClO_4_, or magnesium perchlorate (Mg(ClO_4_)_2_), and MgCl_2_-enriched PBS for samples containing CaCl_2_ or calcium perchlorate (Ca(ClO_4_)_2_). Because cell death occurred at higher temperatures during plating, especially in Ca^2+^ containing samples, plating for all experiments described in this study was carried out rapidly at cold temperatures. The NaCl/MgCl_2_-enriched PBS was precooled to −15°C/−30°C and agar plates to 4°C.

### 2.4. Freeze/thaw cycle experiments

For investigating the effect of dissolved salts on cell survival when subjected to multiple freeze/thaw cycles, we incubated *P. halocryophilus* at 25°C for 1 week in six individual vials. Three of them contained 10 mL of DMSZ growth medium #92 (with no additional NaCl), while the other three samples contained additionally 10 wt% NaCl. After incubation, all samples where repeatedly frozen at −50°C, stored at this temperature between 1 and 3 days, and thawed at room temperature until the unfrozen sample reached 20°C, which took ∼2 h. After taking an aliquot from each sample for CFU determination, the samples were frozen again. These freeze/thaw cycles were repeated up to 70 times, and the survival was tested intermittently. The results for samples with the same growth media composition were averaged, and the standard deviation was calculated.

## 3. Results

### 3.1. Microbial survival rates in chloride brines

**FIG. 2.**
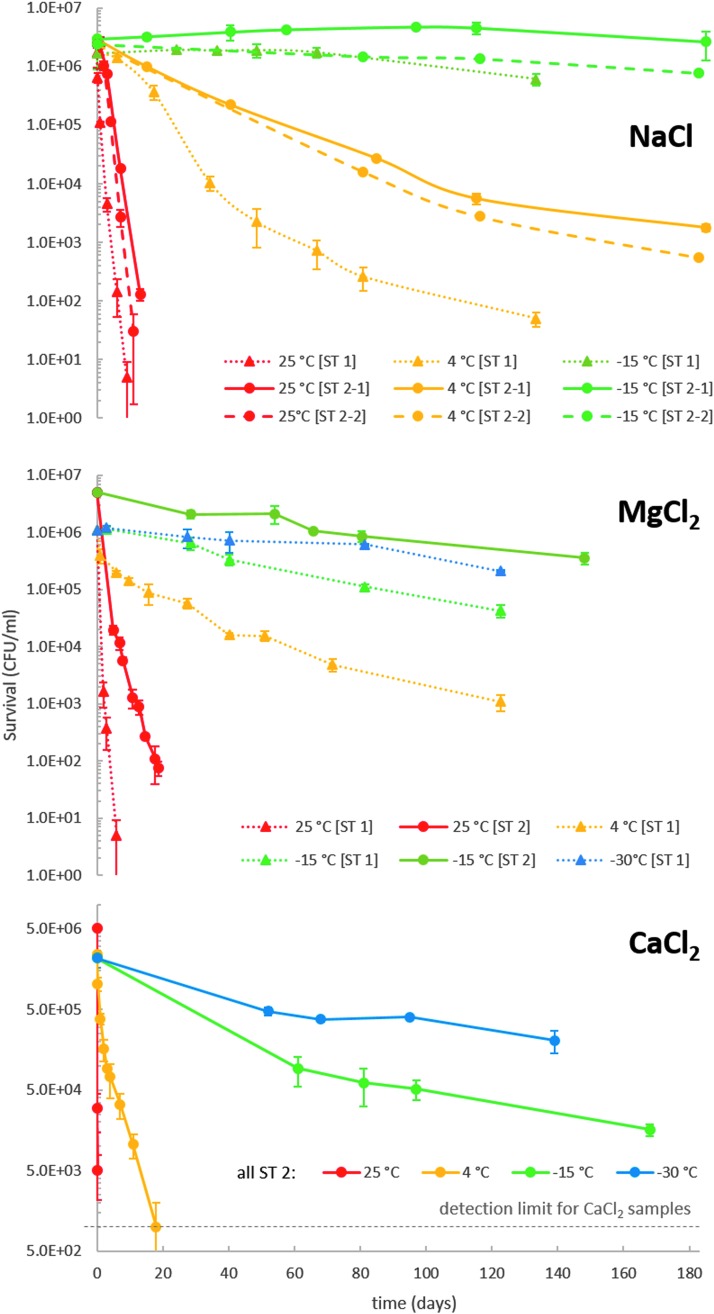
Survival rates of *P. halocryophilus* in eutectic Cl^*−*^ samples. Initial cell cultures were incubated for 4 days (ST 1) or 7 days (ST 2) at 25°C in growth medium containing 10 wt% NaCl before mixing them with the salt solution. CFUs were obtained as biological duplicates. Detection limit for CaCl_2_ containing samples at 10^3^ CFU/mL results from the dilution factor of 3 that is necessary to decrease the Ca^2+^ concentration on the agar plate sufficiently for colony growth to occur.

The survival rates of *P. halocryophilus* in eutectic Cl^−^ samples were significantly increased when the samples were kept at lower temperatures (Fig. 2). For example, if *P. halocryophilus* was left in NaCl containing samples at room temperature, all cells died within 2 weeks, while their survival was substantially increased at 4°C, and nearly no CFU reduction occurred at −15°C. Two samples of ST 2 were investigated for the NaCl system to confirm reproducibility. Samples of ST 2 had slightly higher starting cell numbers in all cases studied. However, survival rates of ST 1 and ST 2 samples were similar, although the curve for the NaCl ST 1 sample at 4°C had a steeper slope during the first 40 days but approached the slope of the ST 2 curves afterward.

The cell survival results for the MgCl_2_ containing samples were very similar to those of NaCl, but at −15°C and −30°C there appears to be a slow reduction of surviving cells. The survival rates of *P. halocryophilus* in CaCl_2_ containing samples at 25°C and 4°C were significantly lower than those containing NaCl or MgCl_2_. In contrast, survival rates at subzero temperatures were comparable to the MgCl_2_ system, that is, cells were dying slower at these lower temperatures.

### 3.2. Microbial survival rates in perchlorate brines

**FIG. 3.**
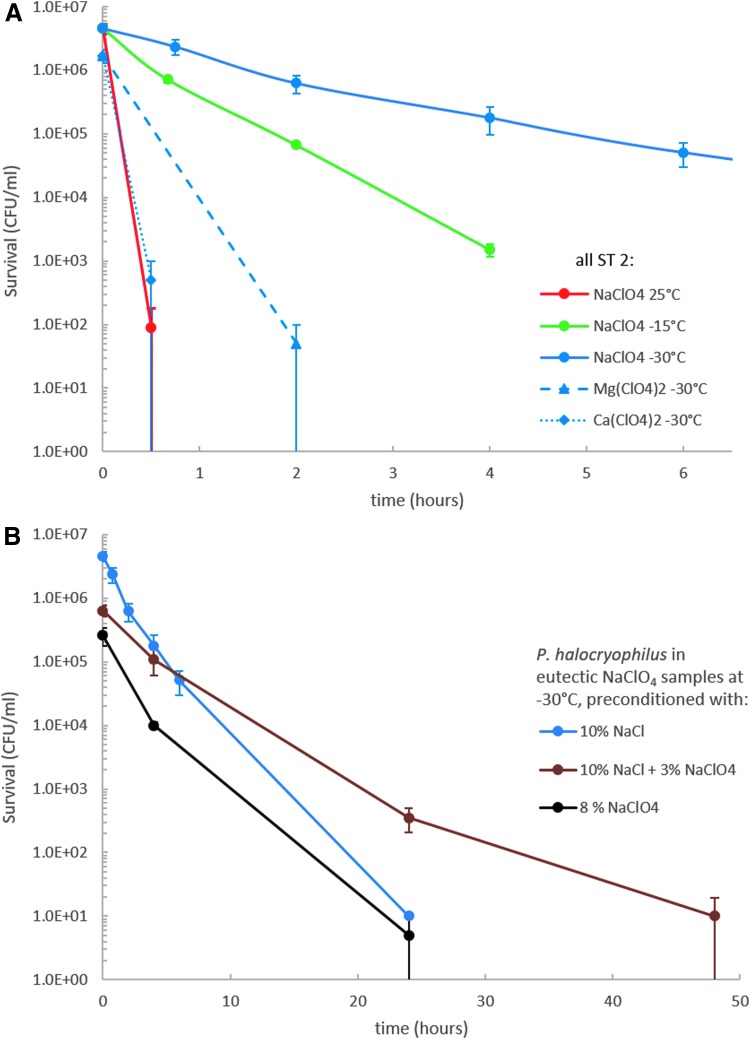
**(A)** Survival rates of *P. halocryophilus* in ClO_4_^*−*^ samples. Initial cell cultures were incubated for 7 days at 25°C in growth medium containing 10 wt% NaCl before mixing them with the salt solution **(B)**. Effects of different preconditioning methods at *−*30°C. Before mixing them with the salt solution, the initial cell cultures were incubated for 7 days at 25°C in growth medium containing salts as indicated in the figure legend. CFUs were obtained in biological duplicates.

The survival rates of *P. halocryophilus* in eutectic ClO_4_^−^ samples (Fig. 3) were orders of magnitude lower than in Cl^−^ samples (Fig. 2). Although survivability at lower temperatures in NaClO_4_ samples increased, the survival rate was generally so low that even at −30°C, few cells survived for only 1 day (Fig. 3A, B). For Mg(ClO_4_)_2_ and Ca(ClO_4_)_2_ containing samples, survival was even lower, where CFU detection was only possible for samples stored at −30°C, and none was detected for samples kept at higher temperatures.

We increased the NaClO_4_ concentration in the growth media to determine whether ClO_4_^−^ preconditioning of *P. halocryophilus* could enhance survival in eutectic ClO_4_^−^ samples. It was found that *P. halocryophilus* can grow in the presence of up to at least 10 wt% NaClO_4_ (with no additional NaCl in the growth medium) or up to 10 wt% NaCl +5 wt% NaClO_4_. However, cell growth under these conditions was markedly slower than in ClO_4_^−^-free medium. Thus, for the preconditioning experiments we used cells preconditioned with 8 wt% NaClO_4_ or with 10 wt% NaCl +3 wt% NaClO_4_ (Fig. 3B). Nevertheless, in these cases, cells grew slower than in the experiments with 10 wt% NaCl in the growth media, which resulted in a lower starting cell number.

Due to the slower growth rates in ClO_4_^−^ containing media, the cells should still be in the exponential growth phase after 7 days of incubation. We found that changing the preconditioning salt from NaCl to NaClO_4_ did not increase the survivability in ClO_4_^−^ containing samples. However, increasing the total salt concentration by adding 3 wt% NaClO_4_ on 10 wt% NaCl resulted in a slight increase in survival. Cells in these samples doubled their maximum survival time from ∼1 day in samples containing either 8 wt% NaClO_4_ or 10 wt% NaCl to 2 days in samples containing 3 wt% NaClO_4_ + 10 wt% NaCl.

### 3.3. Arrhenius plot

**FIG. 4.**
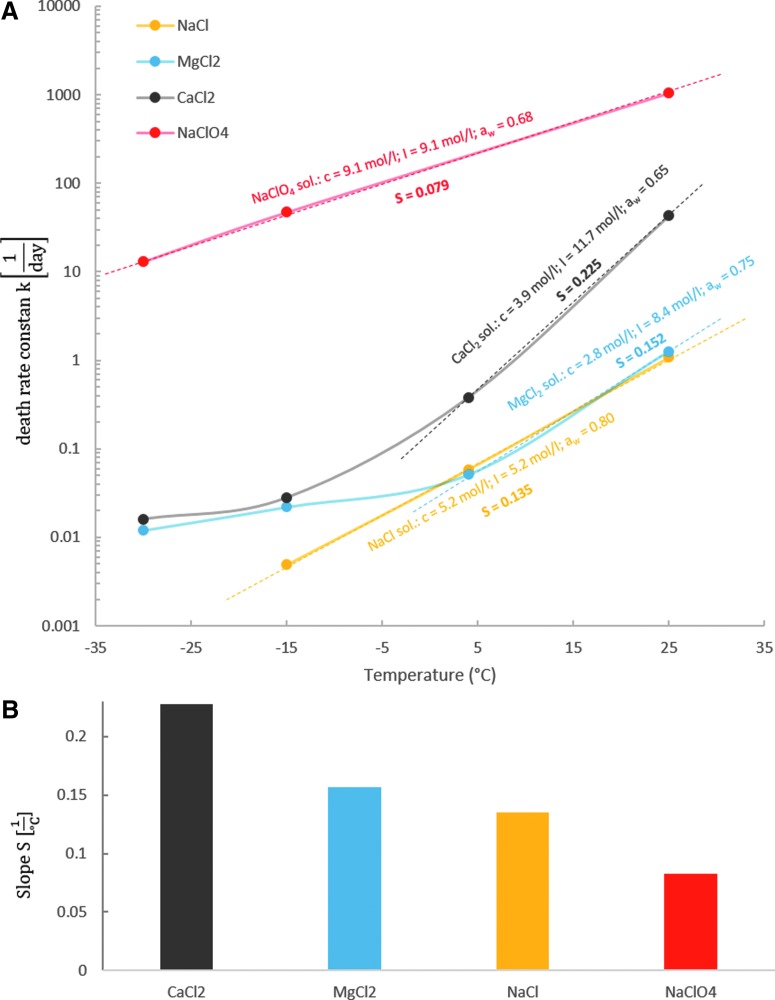
**(A)** Arrhenius-type plot for all Cl^*−*^ samples and NaClO_4_, including slopes (S) for linear parts of the curves and molar concentrations (c), water activities (a_w_), and ionic strengths (I) for all samples. **(B)** Slopes (S) of the steeper curve parts plotted as bar charts.

For a better comparison of the temperature dependences of cell survival in different Cl^−^ and ClO_4_^−^ containing samples, the data were plotted as an Arrhenius-type graph, with the slopes of the survival rate-fitted lines for all Cl^−^ and NaClO_4_ containing samples (values for same salt/temperature combinations were averaged) plotted logarithmically against the temperature of the sample (Fig. 4A). As the slope (S) of these curves is the crucial parameter for evaluating the extent to which survival is increased with lowering temperature, the slope values for each curve were plotted as well (Fig. 4B).

The slopes for the Cl^−^ containing samples, especially for MgCl_2_ and CaCl_2_, flatten below death rate constants of about 0.1 day^−1^. However, it has to be kept in mind that the death rates are on a logarithmic scale, and therefore, the flattening might only be the result of approaching a nonlethal state, that is, a death rate of zero. Therefore, only the steeper slops of the curves toward higher temperatures were compared, as given in Figure 4B.

### 3.4. Microbial survival rates during freeze/thaw cycles

**FIG. 5.**
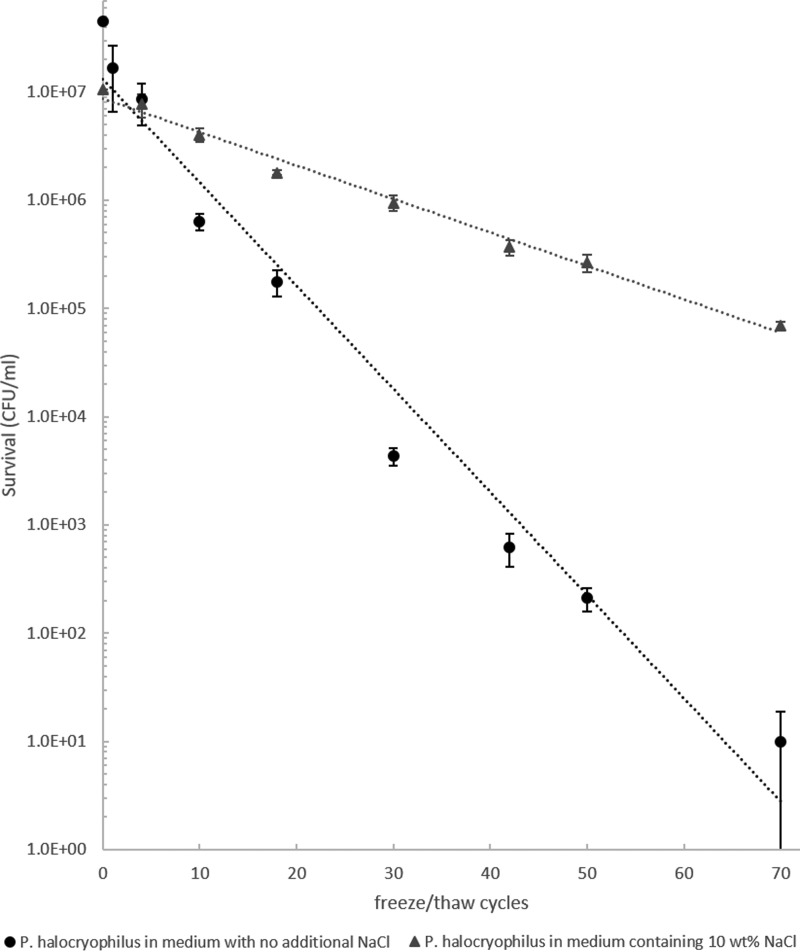
Survivability of *P. halocryophilus* during freeze/thaw cycles. Cells were incubated for 7 days at 25°C in growth medium containing either no additional salt (black circles) or 10 wt% NaCl (gray triangles) before subjecting them to freeze/thaw cycles. CFUs obtained from biological triplicates.

*P. halocryophilus* survived repeated freeze/thaw cycles more readily if the growth medium contained additional NaCl. Without NaCl, the CFU reduction is 20% per freeze/thaw cycle, whereas an addition of 10 wt% NaCl lowered the death rate to 7% per freeze/thaw cycle (Fig. 5). Cells in the salt-free samples survived up to 70 freeze/thaw cycles, while extrapolation of the death rate curve for the samples containing 10 wt% NaCl reveals that cells in these samples could survive up to ∼200 freeze/thaw cycles.

## 4. Discussion

We have shown that survival of *P. halocryophilus* is significantly lower in eutectic ClO_4_^−^ samples than in Cl^−^ containing samples at all investigated temperatures, although ionic strength and water activities at 25°C are similar, for example, for CaCl_2_ and NaClO_4_ samples (Table 1 and Fig. 4A). Moreover, the water activity should not change markedly when lowering the temperature since it has been shown to remain reasonably constant at subzero temperatures for solutions containing Cl^−^ (Fontan and Chirife, 1981) and ClO_4_^−^ (Toner and Catling, 2016). Furthermore, the oxidizing ability of ClO_4_^−^ is negligible in solutions at these low temperatures (Brown and Gu, 2006). Thus, other ion-specific properties must be responsible for the differences in the inhibitory effects of the ClO_4_^−^ and Cl^−^ containing samples.

In addition, we demonstrated that the survival of *P. halocryophilus* cells in eutectic Cl^−^ and ClO_4_^−^ samples increases systematically with decreasing temperatures. The Arrhenius plot (Fig. 4) indicates that this correlation is more significant in Cl^−^ containing samples. The slope for the CaCl_2_ containing samples (0.225°C^−1^) is more than 2.5-fold steeper than for the NaClO_4_ containing samples (0.079°C^−1^), which means that survivability in the CaCl_2_ samples is increased by lowering temperature to a significantly higher extant than in the NaClO_4_ samples. The slopes for MgCl_2_ (0.152°C^−1^) and NaCl (0.135°C^−1^) containing samples lie between those of NaClO_4_ and CaCl_2_.

The slow decrease of the death rate constant in the NaClO_4_ containing samples with decreasing temperature is caused by the normal temperature dependence of all chemical reactions (including cell damaging reactions) described by the Arrhenius equation. The steeper slopes for the Cl^−^ samples indicate an additional effect on the decrease of death rates with lowering the temperature.

We propose that the main reason for this difference in the temperature dependence of the cell survival in Cl^−^ and ClO_4_^−^ containing samples is the increase of size and stability of hydration spheres around the ions in the Cl^−^ brines at lower temperatures. Previous studies have shown that with decreasing temperatures the hydration number around cations such as Ca^2+^ increases (Zavitsas, 2005) and that the first hydration sphere around Na^+^ in NaCl solutions becomes more rigid (Gallo *et al.*, 2011). Furthermore, X-ray and neutron diffraction studies have shown that a decrease in temperature results in the first hydration shell of Cl^−^ ions becoming gradually more structured and a second hydration sphere forming (Yamaguchi *et al.*, 1995). Data from the method of integral equations reveal a strengthening of the hydrogen bonding between Cl^−^ and water molecules in the first hydration shell at lower temperatures (Oparin *et al.*, 2002).

These results demonstrate that lowering the temperature in Cl^−^ containing samples increases the stability and size of hydration spheres around the dissolved ions, known to reduce the permeability of ions through cell membranes (Degrève *et al.*, 1996; Jahnen-Dechent and Ketteler, 2012). Hence, we conclude that a reduced ion permeability caused by larger and more stable hydration spheres minimizes the toxicity of the extracellular high ion concentration. Therefore, cell survivability in low-temperature Cl^−^ brines is increased over the extent of the normal Arrhenius-like temperature dependence.

In contrast to cations and Cl^−^ ions, ClO_4_^−^ ions do not tend to form stable hydration shells (Neilson *et al.*, 1985; Lindqvist-Reis *et al.*, 1998). The reason for the small size and low stability of hydration shells around ClO_4_^−^ is its large ionic radius and its low electrical charge with an even distribution over the entire anion (Brown and Gu, 2006) resulting in weak hydrogen bonds and one of the lowest hydration energies of common inorganic anions (Moyer and Bonnesen, 1979). The low tendency of ClO_4_^−^ ions to form stable hydration spheres at any temperature presumably correlates with a constant cell membrane permeability and is a reasonable explanation for the observed low survival rate increase in ClO_4_^−^ containing samples with lower temperatures.

Finally, the higher membrane permeability at all investigated temperatures explains the general low survivability of cells in ClO_4_^−^ verses Cl^−^ containing samples. However, several other structural factors may play a significant role as well, for example, the formation of chloro-complexes in CaCl_2_ containing samples (Phutela and Pitzer, 1983; Wang *et al.*, 2016), ion pair formations (Fleissner *et al.*, 1993; Smirnov *et al.*, 1998), molecular mimicry (Cianchetta *et al.*, 2010), or the reported formation of a crust around *P. halocryophilus* cells at low temperatures, consisting of peptidoglycan, choline, and calcium carbonate (Mykytczuk *et al.*, 2016) that might provide protection against Cl^−^ but not ClO_4_^−^.

The freeze/thaw experiments have shown that the survivability of cells during freezing and thawing processes increases when NaCl is present. Studies have argued that the formation of large water crystals during freezing might be destructive to cell membranes and might even grow larger during thawing due to migratory recrystallization (Mazur, 1960). Greater amounts of large water crystals should only form in the salt-free samples, because in the salt-rich samples, pure water crystals are formed during freezing only until the solution under the ice layer reaches the eutectic composition. After that point, eutectic freezing results in very small water ice and salt hydrate particles, which potentially could be physically less harmful to the cells.

Another lethal effect during the freezing process might be the osmotic shock resulting from the increasing solute concentration in the remaining liquid solution (Harrison, 1956). It is reasonable to assume that the decreased water activity, as a result of the enhanced solute concentration in the growth media during the freezing process, is less harmful to bacteria that were already preconditioned with 10 wt% NaCl during incubation. Furthermore, studies have shown that a heat or cold shock treatment of *Deinococcus radiodurans* cells increases their survivability against freeze/thaw cycles (Airo *et al.*, 2004), hence an exposure to higher salt concentrations may result in a similar stress response in *P. halocryophilus* and a higher tolerance against freeze/thaw cycling. The beneficial effect of NaCl during the freeze/thaw process has been described in previous studies (*e.g.*, Calcott and Rose, 1982).

In contrast, other studies have shown the opposite trend, that the presence of NaCl decreases the percent of surviving bacteria during freeze/thaw cycles (Postgate and Hunter, 1961; Nelson and Parkinson, 1979). However, these bacteria are not known to be halotolerant and therefore might suffer more under increased osmotic stress than *P. halocryophilus* does. In the future, freeze/thaw experiments with halophilic microorganisms such as *P. halocryophilus* should also include other types of salts in the growth medium to test their influence on cell survivability in comparison with NaCl.

On Mars, NaCl has been detected globally, and especially at high levels by remote sensing in the Southern Highlands (Osterloo *et al.*, 2008). Perchlorates have been detected at the Phoenix Lander and the Curiosity Rover sites and are likely global in extent (Clark and Kounaves, 2016). These salts have also been suggested to be part of the brines associated with the recurrent slope lineae (RSL) (Ojha *et al.*, 2015). However, more recent studies have argued that only small amounts of water might be present within the RSL (Edwards and Piqueux, 2016) and that the darkening of the RSL might only be a result of a rewetting process of former flows of salty water (Heinz *et al.*, 2016). Furthermore, it has also been suggested that RSL may be the result of granular flows where water plays no, or only a subordinate, role (Dundas *et al.*, 2017).

However, in general, the ubiquitous presence of hygroscopic salts and of water in the form of ice on the poles or in the subsurface or as gas in the atmosphere makes the existence of cold, highly concentrated brines conceivable. Such brines could develop through deliquescence or at salt-ice contacts (Fischer *et al.*, 2014), being temporally stable at the surface of Mars and perhaps permanently stable in the subsurface as briny groundwater (Burt and Knauth, 2003; Martínez and Renno, 2013).

Our data reveal that microorganisms resident in such brines could survive significantly longer at subzero temperatures than previously thought, and they might even thrive in slightly diluted brines as has been shown for *P. halocryophilus* in a 19 wt% NaCl solution (Mykytczuk *et al.*, 2012). As temperatures on Mars change throughout the day and the seasons, it is conceivable that temperatures drop temporally below the eutectic temperature of the brine. Our freeze/thaw experiments demonstrate that the freezing and thawing of cells in eutectic brines would be less lethal than freezing and thawing in salt-free water.

## 5. Conclusion

We have shown enhanced microbial survival in subzero eutectic Cl^−^ brines compared with their warmer analogues. Based on the results, the best hypothesis is that the increase in size and stability of hydration shells around ions at lower temperatures reduces osmotic and chaotropic stress factors for microbial organisms.

Although *P. halocryophilus* grew even in the presence of 10 wt% NaClO_4_, higher ClO_4_^−^ concentrations lower survival rates significantly even at subzero temperatures. It appears that the decreased capability of ClO_4_^−^ ions to form stable hydration spheres causes the high toxicity of eutectic ClO_4_^−^ solutions and the lower temperature dependence of cell survival compared with Cl^−^ brines. Furthermore, we have shown that the presence of salts such as NaCl increases the survivability during freeze/thaw processes. This has broad implications for the habitability of some extreme environments on Earth and the potential habitability of Mars.

## Abbreviations Used

CFUs: colony forming units
PBS: phosphate-buffered saline
RSL: recurrent slope lineae
ST: sample type
